# In-Line Phase Contrast Imaging of Hepatic Portal Vein Embolization with Radiolucent Embolic Agents in Mice: A Preliminary Study

**DOI:** 10.1371/journal.pone.0080919

**Published:** 2013-12-04

**Authors:** Rongbiao Tang, Wei Huang, Fuhua Yan, Yong Lu, Wei-Min Chai, Guo-Yuan Yang, Ke-Min Chen

**Affiliations:** 1 Department of Radiology, Rui Jin Hospital, Shanghai Jiao Tong University School of Medicine, Shanghai, People’s Republic of China; 2 Neuroscience and Neuroengineering Center, Med-X Research Institute, Shanghai Jiao Tong University, Shanghai, People’s Republic of China; Glasgow University, United Kingdom

## Abstract

It is crucial to understand the distribution of embolic agents inside target liver during and after the hepatic portal vein embolization (PVE) procedure. For a long time, the problem has not been well solved due to the radiolucency of embolic agents and the resolution limitation of conventional radiography. In this study, we first reported use of fluorescent carboxyl microspheres (FCM) as radiolucent embolic agents for embolizing hepatic portal veins. The fluorescent characteristic of FCM could help to determine their approximate location easily. Additionally, the microspheres were found to be fairly good embolizing agents for PVE. After the livers were excised and fixed, they were imaged by in-line phase contrast imaging (PCI), which greatly improved the detection of the radiolucent embolic agents as compared to absorption contrast imaging (ACI). The preliminary study has for the first time shown that PCI has great potential in the pre-clinical investigation of PVE with radiolucent embolic agents.

## Introduction

Preoperative portal vein embolization (PVE) is an effective modality to induce hepatic hypertrophy by obstructing the selective portal vein supplying the diseased segment of the liver [1]–[4]. In order to prevent blockage in the wrong region, it is quite essential to determine the distribution of the embolic agents inside target liver during and after the embolization. Clinically, gelatin sponge (GS) and polyvinyl alcohol particles (PVA) are the most commonly used embolic agents [5], [6]. However, GS and PVA are low-absorption materials which are hardly visualized by conventional radiography. Therefore, embolic agents are always injected by combining them with iodine contrast agent to enhance image contrast. Nevertheless, iodine-enhanced method only indirectly shows the embolization site, and can not accurately verify the distribution of embolic agents. Additionally, it is still difficult to show fine embolized vessels with a diameter of 200 µm or less by conventional angiography due to the limitation of spatial resolution [7].

To overcome the challenges, novel imaging method should be applied. Currently, synchrotron radiation (SR) phase contrast imaging (PCI) has been widely utilized to provide excellent image contrast for soft tissues [8]–[11]. PCI, utilizing the phase shift, can produce higher contrast images than absorption contrast imaging (ACI) [12], [13]. Also, PCI is considered as a powerful preclinical imaging modality to observe fine structures with its resolution higher than any available clinical radiography [14], [15]. Using PCI, hepatic vessels down to micron level can be clearly shown without using contrast agents [16], [17]. The values of these applications raise the possibility of using PCI for clearly imaging low-absorption embolic materials.

In this study, GS and PVA were imaged by SR imaging. PCI and ACI were performed and compared. We evaluated the feasibility of using PCI for imaging PVE with radiolucent fluorescent carboxyl microspheres (FCM).

## Materials and Methods

### Sample Preparation

All experiments were conducted in accordance with the guidelines established and approved by Shanghai Jiao Tong University's Institutional Animal Care and Use Committee. 6 male ICR mice were anesthetized using an intraperitoneal injection of ketamine (100 mg kg^−1^) and xylazine (10 mg kg^−1^). The main portal trunk was dissected, and then punctured with a thin PE-50 catheter through a midline laparotomy. Then PVE was performed by injecting 100 FCM (in 0.1 ml PBS) into the portal vein via the catheter attached to a 1 ml syringe. 5 minutes after the PVE, mice were sacrificed by cervical dislocation under anesthesia. The livers were harvested and placed in 4% formaldehyde solution. The numbers of FCM in main lobes of liver were counted under fluorescence microscope. Data were expressed in mean ± standard deviation. Three non-dehydrated livers were randomly chosen to scan by phase contrast CT imaging. For imaging dehydrated livers, the other three livers were placed in a 4% formaldehyde solution for 72 hours, dehydrated with 100% ethanol for 48 hours, and then placed in the air for 2 hours.

### SR Imaging Parameters

**Figure 1:**
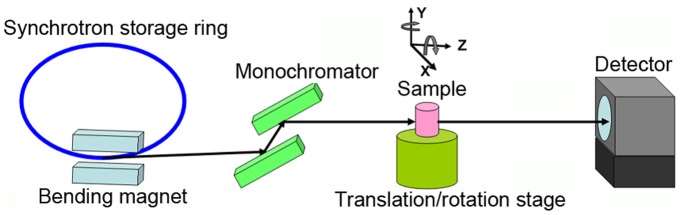
Schematic of the experimental setup for in-line phase contrast imaging at BL13W in SSRF. The sample-to-detector distance has a changeable range of 8 m.

Imaging was performed at the BL13W1 beamline in Shanghai Synchrotron Radiation Facility (SSRF, China). X-rays were derived from a 3.5 GeV electron storage ring. The beamline covered an energy range of 8 to 72.5 keV. X-Ray was monochromatized at 19 keV energy using a double-crystal monochromator with Si(111) and Si(311) crystals. The energy resolution was △E/E<5×10^−3^. The transmitted x-rays were first converted to visible light by a scintillator consisting of a 100 µm thick CdWO_4_ cleaved single crystal, and then captured by a CCD camera with the pixel size of 3.7 µm. (Photonic Science, UK). Samples were positioned on a translation/rotation stage at a distance of 34 m from the synchrotron source. The distance between the sample and the detector had a changeable range of 8 m (Fig. 1).

### Comparison between ACI and PCI

Clinically utilized 150–350-µm GS (Eric Kang, China) and 90–180-µm PVA (PVA-100, Cook) were purchased for imaging. FCM (ACMEmicrospheres, USA) were used for hepatic PVE. FCM had a mean diameter of 100 µm, ranging from 90 to 105 µm. ACI and PCI were performed with the same imaging parameters except sample-to-detector distance (d = 1 cm and 60 cm, respectively). The distance was changed by moving the CCD camera on a rail. Relative densities were evaluated by line profile analysis via Image-Pro Plus 6.0.

### Phase Contrast CT Imaging

1000 projection images were obtained from each sample over 180° in rotation steps of 0.18°. The projections were recorded with sample-to-detector distance of 60 cm and exposure time of 1 s. The raw data were processed by applying the filtered back projection (FBP) algorithm with PITRE software [18]. 3D phase contrast reconstructed images were acquired by using the Amira 5.2 software (Mercury Computer Systems, USA).

## Results

### SR Imaging of GS and PVA

**Figure 2:**
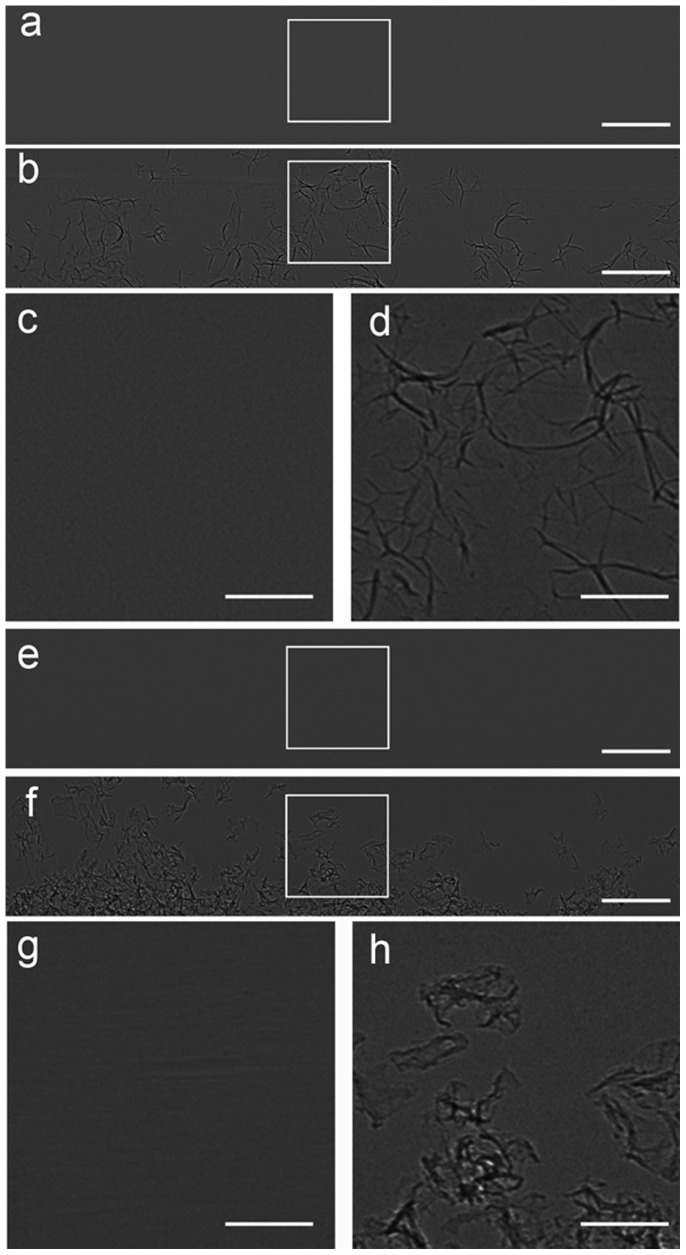
SR images of GS (a-d) and PVA (e-h) at 19 keV with two sample-to-detector distances of 1 cm (a,e) and 60 cm (b,f). (c), (d), (g) and (h) are magnified images of the region in a white box in (a), (b), (e) and (f), respectively. The GS and PVA can be clearly displayed on phase contrast images (b,d,f,h), but not on absorption contrast images (a,c,e,g). GS and PVA were exposed to the air. The pixel size was 3.7 µm×3.7 µm. The exposure time was 1 s. Scale bars, 500 µm (a,b,e,f) and 200 µm (c,d,g,h).

The radiolucent characteristic of GS and PVA was demonstrated in Fig. 2. No distinct contrast between GS or PVA and its surrounding air could be observed on the absorption images (Figs. 2c and g). After adjusting the distance to 60 cm, we were able to clearly visualize the GS and PVA on the phase contrast images (Figs. 2d and h). The two embolic agents were both irregular in shape (Figs. 2b and f).

### Morphological Observation and SR Imaging of FCM

**Figure 3:**
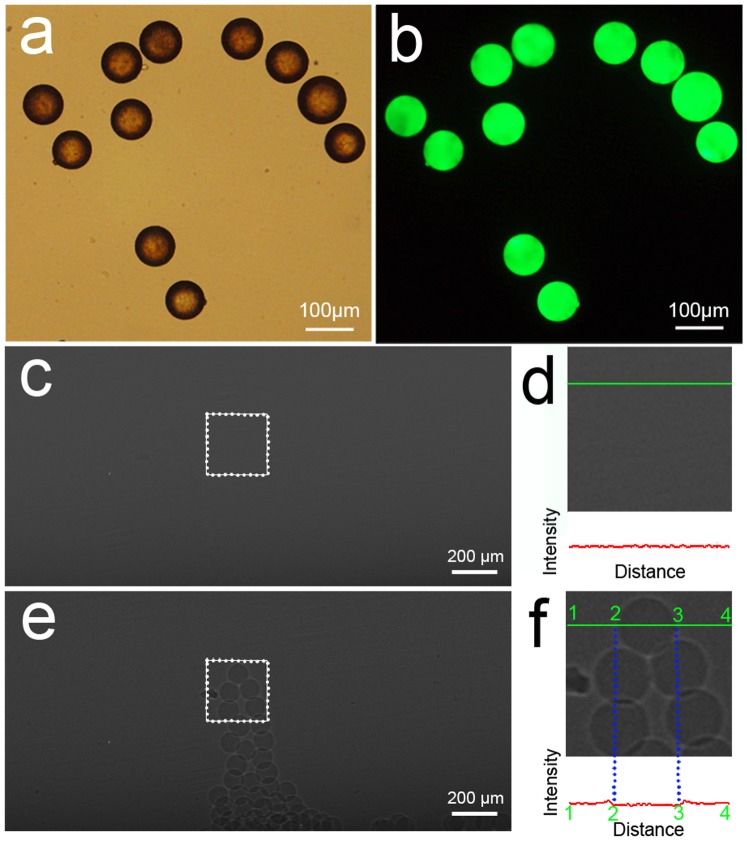
Microscopic observation and SR images of FCM. (a) Optical micrograph shows that FCM are spherical particles with smooth surface. (b) FCM presents bright green fluorescence. (d) and (f) are magnified images of the region in a box in (c) and (e), respectively. The microspheres can be clearly seen on phase contrast image (f), but not on absorption contrast image (d). The intensity values along a straight line (green) were displayed by using line profile analysis (red). Note that the beads caused a reduced change in intensity between the left (2) and right (3) boundaries on the phase contrast image (f). Images were obtained at the energy of 19 keV with two sample-to-detector distances of 1 cm (c) and 60 cm (e). The pixel size was 3.7 µm×3.7 µm; the exposure time was 1 s.

**Figure 4:**
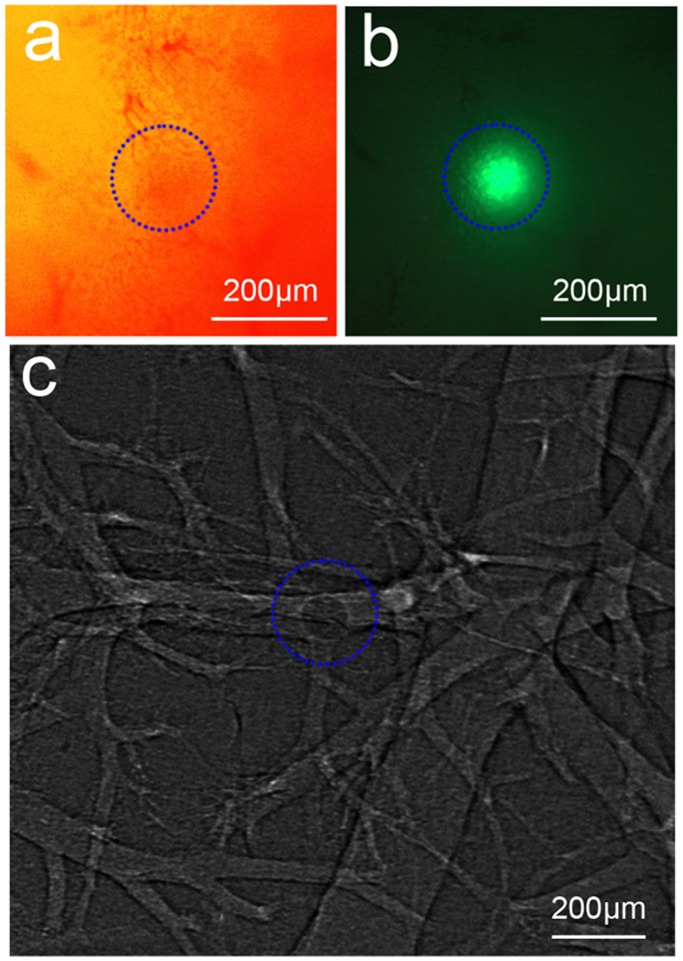
Microscopic observation and PCI of FCM-embolized liver. The fluorescent micrograph (b) shows FCM in the liver more clearly than optical micrograph (a). (c) A FCM can be clearly shown in the portal vein. The blue circles indicate FCM. Image was obtained at the energy of 19 keV with the sample-to-detector distance of 60 cm. The pixel size was 3.7 µm×3.7 µm; the exposure time was 1 s.

FCM have smooth and uniform surface morphology, as confirmed by optical microscopy (Fig. 3a). The microspheres also present bright green fluorescence (Fig. 3b). In Figs. 3c and d, the images obtained by absorption contrast could not reveal the microspheres at all. In comparison, PCI could provide clear visualization of the microspheres (Figs. 3e and f). The beads caused a visible change in intensity around the edges on the phase contrast image (Fig. 3f). The fluorescent micrograph (Fig. 4b) shows FCM in the liver more clearly than optical micrograph (Fig. 4a). Nos. of FCM in left lateral lobe, right middle lobe and left middle lobe were 12.33±3.27, 8.83±1.72 and 6.33±2.34, respectively (Table 1).

**Table 1:** Nos. of FCM in three main lobes of liver.

	No. of FCM	
Lobe of liver	Mean	Standard deviation
Left lateral lobe	12.33	3.27
Right middle lobe	8.83	1.72
Left middle lobe	6.33	2.34

### PCI of Hepatic Embolization

**Figure 5:**
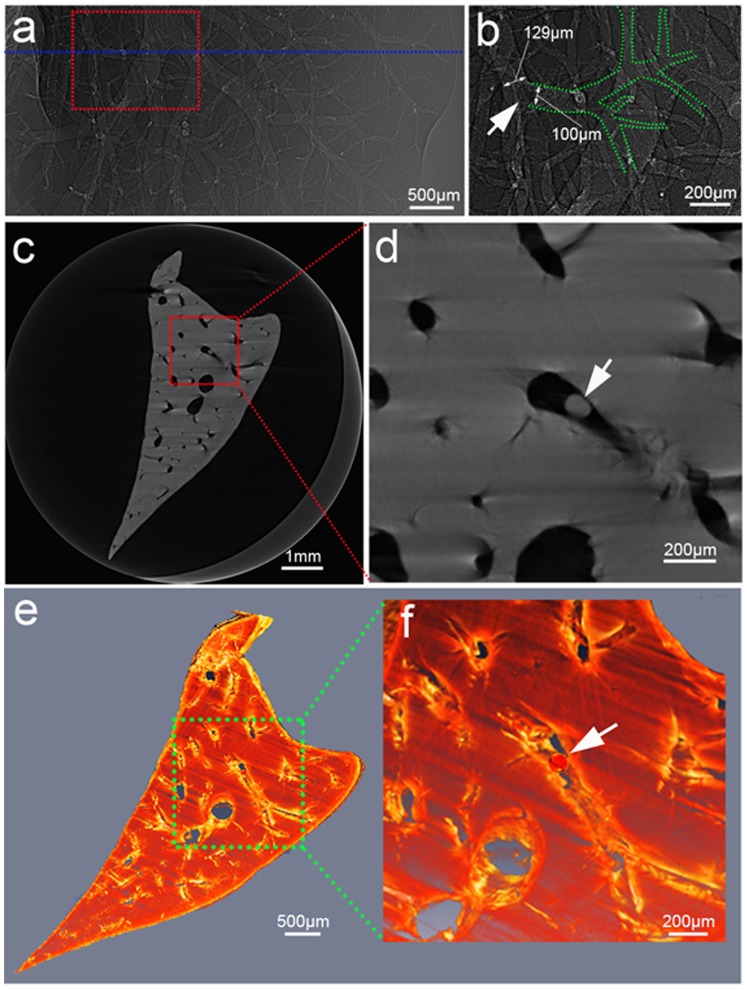
Phase contrast CT imaging of FCM-embolized dehydrated liver. Projection image (a) and reconstructed views (c,e) visibly show the FCM in the embolized portal vein. (b), (d) and (f) are magnified images of the region in a dotted box in (a), (c) and (e), respectively. (b) An about 100-µm portal vein and its branches (outlined in green) are clearly displayed to be embolized by a 100-µm FCM. (c) is the axial slice across the blue line in (a). (f) The 3D rendering shows spatial distribution of the microsphere inside the liver. Arrows indicate FCM. Images were obtained at the energy of 19 keV with the sample-to-detector distance of 60 cm. The pixel size was 3.7 µm×3.7 µm; the exposure time was 1 s.

**Figure 6:**
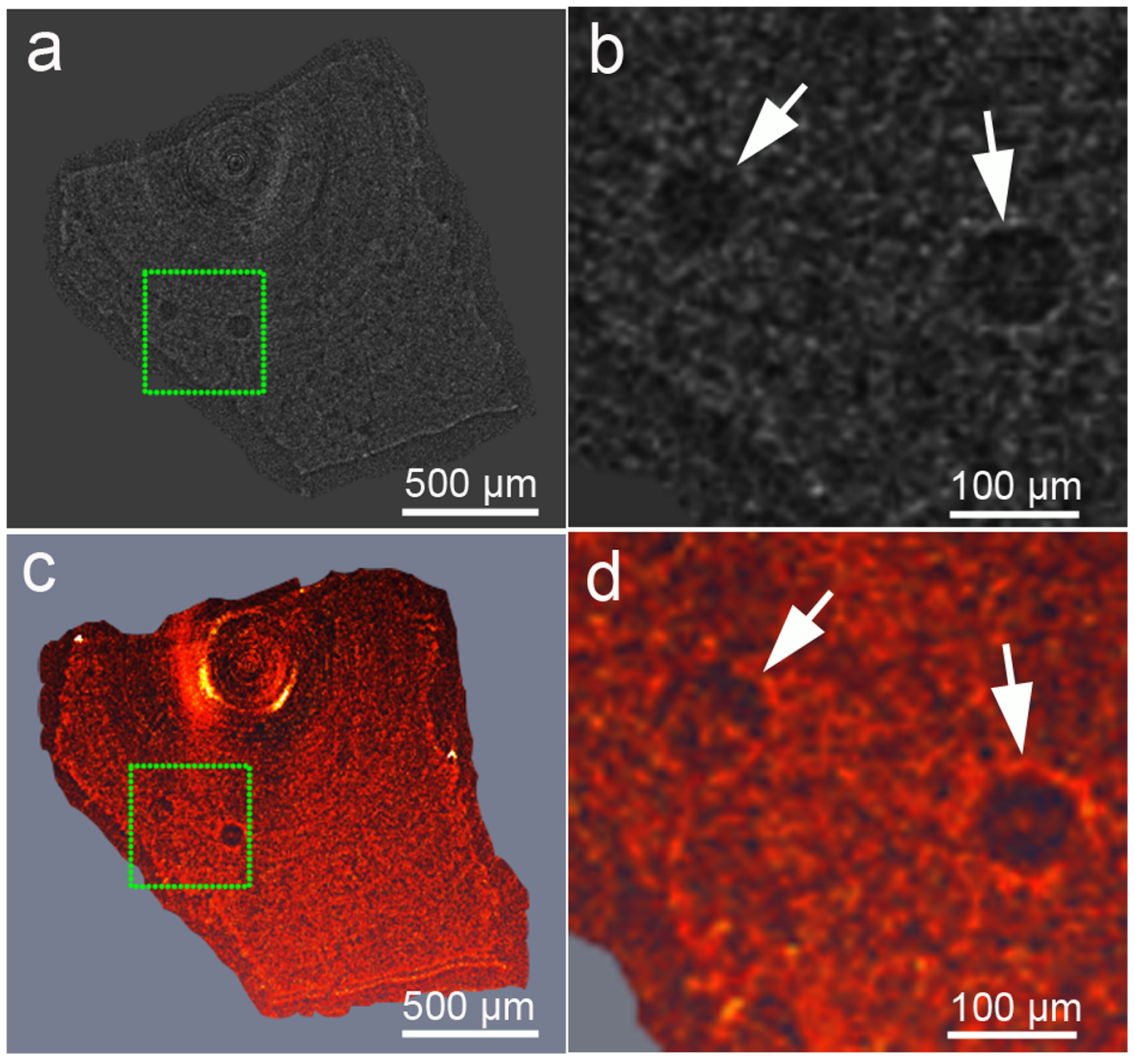
Phase contrast CT imaging of FCM-embolized non-dehydrated liver. Axial (a) and 3D (c) views of FCM in the liver. (b) and (d) are magnified images of the region in a dotted box in (a) and (c), respectively. Note that the phase contrast CT imaging clearly showed the radiolucent FCM in the liver tissues. Arrows indicate FCM. Images were obtained at the energy of 19 keV with the sample-to-detector distance of 60 cm. The pixel size was 3.7 µm×3.7 µm; the exposure time was 1 s.

FCM could be clearly identified inside the occluded vessel in dehydrated livers (Figs. 4c and 5a). In Fig. 5b, an about 100-µm portal vein and its branches are clearly shown to be embolized by a 100-µm FCM. In Figs. 5c and d, FCM can be clearly distinguished from the wall of the vessel on axial view. The spatial distribution of FCM inside target vessels could be clearly revealed by 3D reconstruction application (Figs. 5e and f). The FCM could also be clearly revealed to identify the embolized segment of the non-dehydrated liver (Fig. 6).

## Discussion

In order to prevent postoperative liver insufficiency, PVE is often clinically used to stimulate growth of the non-embolized liver segment [19]–[21]. The effect will be better if the embolic agents selectively block the blood supply that feeds the tumor. Accordingly, good knowledge of embolic spatial distribution is vital to make embolization at the desired liver segment. The present study has for the first time reported that PCI has great potential for pre-clinical PVE investigation with radiolucent embolic agents.

Though the effects of x-ray scatters on the absorption contrast images are significantly reduced by using narrow SR x-ray beam, no distinct contrast between GS or PVA and its surrounding air could be observed by ACI. By increasing the sample-to-detector distance, in-line phase contrast can be obtained for the sample [22], [23]. PCI has been shown to be quite suitable for liver research, such as liver fibrosis [24] and liver cancer [25]. PCI, unlike ACI, depends mainly on phase shift properties to offer greatly improved contrast for low-absorption materials [10], [26], [27]. The phase contrast technique can convert such phase shifts into intensity differences that can be detected directly. The two embolic agents present irregular in shape. However, the irregular shape and variable size of these particles may present a poor correlation of the occlusion level and the particle size [28]. When large particles are properly oriented, they can reach a distal vessel. In addition, the aggregation behavior of the particles may cause blockage of proximal large vessels rather other desired vessels. Nowadays, spherical embolic agents with uniform shape have been developed to overcome the disadvantages of conventional embolic agents [28]–[30]. The microspheres can make a predictable occlusion of vessels according to the particle size selected. Here, we use spherical FCM as radiolucent embolic agents. The fluorescent characteristic of the microspheres can offer the potential for multimodal imaging. Because the difference between the absorption coefficients of FCM and air is small, no evident contrast can be detected by ACI. In comparison, PCI exploits the differences in the refractive index and enables clear visualization of the FCM.

The hepatic vessels were filled with air to replace blood after they were fully dehydrated. On PCI, the phase shifts arising from FCM-air interfaces can generate sufficient image contrast to show the weak-absorption FCM. After the injection, FCM run through vessels, and then stop when they reach vessel of their own size. Thus, the embolization level was directly related to the diameter of injected embolic agents. Many embolic beads were found in three main lobes of liver; still, some FCM retained in main portal vein. In further study, super-selective catheterization technique may be used to concentrate the embolic agents in the targeted hepatic lobe. The phase contrast CT imaging could also provide adequate image contrast to distinguish the FCM from the non-dehydrated liver tissues; consequently, the embolized liver segment could be directly and obviously identified. Besides phase contrast, high spatial-resolution performance is another important characteristic for PCI using SR [31], [32]. The spatial resolution of PCI can arrive at submicron scale, which is much higher than that of conventional angiography using x-ray, CT, and MRI [33]–[35]. On PCI, we could visualize vessels of about one-tenth of the diameter measured by conventional angiography. So PCI has the potential for imaging fine embolized vessels.

In summary, the characteristics of sensitivity to low-absorption materials and high resolution for PCI are very attractive. Compared with ACI, PCI is capable of creating remarkable visibility of radiolucent embolic agents. In addition, phase contrast CT technology can help to noticeably reveal the 3D spatial distribution of low-absorption embolic agents and clearly identify the embolized liver segment. Therefore, PCI can be currently used for pre-clinical evaluation of new embolic agents in animal models; meanwhile, the imaging modality may allow potential medical application if a compact SR x-ray source is employed in the future.
